# Hydroxyurea prevents arterial and late venous thrombotic recurrences in patients with myeloproliferative neoplasms but fails in the splanchnic venous district. Pooled analysis of 1500 cases

**DOI:** 10.1038/s41408-018-0151-y

**Published:** 2018-11-12

**Authors:** Valerio De Stefano, Elena Rossi, Alessandra Carobbio, Arianna Ghirardi, Silvia Betti, Guido Finazzi, Alessandro M. Vannucchi, Tiziano Barbui

**Affiliations:** 1 Fondazione Policlinico Universitario A. Gemelli IRCCS, Roma, Italy; 2 Istituto di Ematologia, Università Cattolica del Sacro Cuore, Roma, Italy; 3 FROM Research Foundation, Papa Giovanni XXIII Hospital, Bergamo, Italy; 4 Division of Hematology, Papa Giovanni XXIII Hospital, Bergamo, Italy; 5 CRIMM-Center of Research and Innovation of Myeloproliferative Neoplasms, Azienda Ospedaliera Universitaria Careggi, and Department Experimental and Clinical Medicine, University of Florence, Firenze, Italy

## Abstract

We collected 1500 patients with myeloproliferative neoplasms (MPN) and arterial or venous thrombosis (935/565), pooling three independent cohorts previously reported. Long-term treatment with antiplatelet drugs or vitamin K-antagonists (VKA) was given to 1391 (92.7%) patients; 975 (65%) patients received hydroxyurea (HU). We recorded 348 recurrences (venous in 142 cases) over 6075 patient-years, with an incidence rate of 5.7 per 100 pt-years (95% CI 5.1–6.4). The site of the first thrombosis predicted the site of recurrence. Independent factors influencing the rate of novel arterial thrombosis were HU (HR 0.67, 95% CI 0.46–0.98), antiplatelet treatment (HR 0.54, 95% CI 0.35–0.82), and VKA (HR 0.58, 95% CI 0.35–0.96). On the contrary, the recurrence of venous thromboses was significantly diminished only by VKA (HR 0.60, 95% CI 0.37–0.95), while HU prevented late but not early recurrences after venous thrombosis at common sites. Of note, we failed to demonstrate a positive effect of HU in the prevention of recurrent splanchnic vein thrombosis. In conclusion, in MPN patients, HU plays a role in the prevention of arterial thrombosis, together with aspirin and VKA, whereas its action in the prevention of recurrent venous thrombosis is uncertain. Such findings call for future studies to optimize and personalize secondary prophylaxis after MPN-related thrombosis.

## Introduction

The principal burden of illness in patients with Philadelphia-negative myeloproliferative neoplasms (MPN) is arterial thrombosis and venous thromboembolism (VTE), which can occur since the very early stages of disease and complicate the course of the follow-up with an elevated rate constant over time^1^; on the other hand, fibrotic or leukemic transformation are late complications. The incidence of either arterial thrombosis or VTE is approximately tenfold higher than in the general population^2,3^.

In MPN patients having experienced a first thrombosis, the rate of recurrence is 4.8% patient-years after an ischemic cerebrovascular event^4^ and 6–7.6% patient-years after a venous event^4–8^. Prevention of thrombosis or re-thrombosis is the major goal of care in MPN patients. However, the efficacy of conventional strategies of secondary antithrombotic prophylaxis is unsatisfactory either using antiplatelet agents after arterial events^4,5^ or vitamin K-antagonists (VKA) after venous events^6–8^.

Three randomized clinical trials demonstrated the efficacy of hydroxyurea (HU) in reducing the rate of thrombosis in high-risk patients with essential thrombocythemia (ET)^9–11^; moreover, in a recent reappraisal of the European Collaborative Low-dose aspirin (ECLAP) study, high-risk patients with polycythemia vera (PV) resulted better protected by HU than phlebotomies alone^12^. However, there is some evidence that the benefit of HU is somewhat vessel site-related. In the PT-1 trial, the HU arm had an excess of venous thromboses in respect to the anagrelide arm^10^. Moreover, in a retrospective cohort of 494 MPN patients with previous thrombosis, HU was effective in preventing recurrences in patients with a first arterial event but not in those with a first venous event^5^. On the other hand, in a retrospective cohort of 597 MPN patients with ischemic stroke or TIA, HU resulted in a strong protective factor for the development of recurrent ischemic stroke^4^. Finally, we recently investigated more thoroughly the PV patients recruited in the ECLAP cohort, showing that HU was more effective than phlebotomy in preventing either first or recurrent arterial thromboses, but not venous thromboses^13^.

To investigate further on whether different antithrombotic or cytoreductive agents can play different roles in the prevention of recurrent thrombosis in MPN patients, we took advantage of several large databases built in the last decade, pooling a cohort of 1500 patients with MPN and previous thrombosis.

## Patients and methods

In the last decade, three studies specifically aimed to investigate the rate of recurrent thrombosis, and the effects of secondary prophylactic treatments have been conducted by the Italian Network on MPN. The details of the recruitment criteria have been reported elsewhere^4,5,7,8^. Briefly, the participants were asked to identify, among all consecutive patients with MPN referred to their centers, those who had suffered from thrombosis. The index event had to be concurrent with MPN diagnosis, or in 2 years before, or occurring during a previously diagnosed MPN disease. The major thrombotic events of interest were ischemic stroke, transient ischemic attacks, acute myocardial infarction, unstable angina pectoris, peripheral arterial thrombosis, retinal artery or vein occlusion, deep venous thrombosis (including thrombosis of cerebral and splanchnic veins), and pulmonary embolism. Acute coronary syndrome encompassed acute myocardial infarction as well as unstable angina pectoris. Cerebrovascular disease encompassed ischemic stroke as well as transient ischemic attacks. Splanchnic venous thrombosis included occlusion of hepatic, portal, mesenteric, and splenic veins. The diagnosis of a first or recurrent major thrombotic event was accepted only if objectively proven as previously described^4,5,7,8^. For each patient, the following information was recorded: demographic data, WHO diagnosis, location of thrombosis, method of objective diagnosis, presence of microvascular disturbances or constitutional symptoms, mutational profile, results of the laboratory investigation for thrombophilia, full blood count at diagnosis and at thrombosis, and the presence of constitutional risk factors, including history of previous thrombosis before the index event, smoking habit, hypertension and dyslipidemia, diabetes, and risk factors for cardiogenic embolism or vascular embolism. Moreover, the presence of circumstantial risk factors at the time of an episode of VTE, such as surgery, pregnancy, puerperium, oral contraceptive intake, hormone replacement therapy, trauma, leg cast and prolonged bed immobilization, and long travel, was also recorded; in the absence of these risk factors, VTE was considered unprovoked. Finally, data regarding cytoreductive or antithrombotic treatment after thrombosis, the duration of the treatment, and the reasons for discontinuation of the therapy were recorded. Major bleeding events were also recorded^4,5,7,8^.

The first study recruited MPN patients consecutively diagnosed from January 1985 to December 2005^5^, whereas the other two studies recruited patients with a diagnosis of MPN carried out from January 2005^4,7,8^. However, a careful comparison of the database checked the patients diagnosed in 2005, and no patient resulted to be included twice.

The first study was conducted in the frame of the GIMEMA (Gruppo Italiano Malattie Ematologiche dell’Adulto) and included 494 patients with PV or ET and either arterial (*n* = 338) or venous thrombosis (*n* = 156); 27 of them had a diagnosis of superficial vein thrombosis and were excluded from further analysis^5^. The other two studies were conducted in the frame of the ELN (European LeukemiaNet). One recruited 436 MPN patients with VTE, including 206 patients with VTE at common sites^7^, 181 patients with splanchnic vein thrombosis^8^, and 35 patients with cerebral vein thrombosis so far unpublished. Finally, the second ELN study recruited 597 MPN patients with TIA (*n* = 270) or ischemic stroke (*n* = 327)^4^.

The three studies were designed by the same principal investigators (VDS and TB) employing the same criteria of inclusion over time, so that the individual patient data from the original database were considered eligible to be combined across the studies.

### Statistical methods

Differences in proportions were estimated by the Fisher’s exact test (statistical significance threshold set at *p* < 0.05). The annual incidence rate of an event that occurred during the follow-up was calculated by dividing the number of events by the total number of patient-years. The probability of recurrence as a function of time was estimated using the Kaplan–Meier method by analyzing the interval between the initial thrombosis and a recurrent thrombotic event (uncensored observations), or the duration of time until death, or the time elapsed until the patient’s final visit to the center (censored observations). The probability of recurrence was compared between the groups using the log-rank test (statistical significance threshold set at *p* < 0.05), and the relative risk of recurrence was estimated as a hazard ratio (HR) with a 95% confidence interval using a Cox proportional hazards regression model. The HR was adjusted taking recurrence as the dependent variable and with covariates being sex, age at the time of the index thrombotic event (less than or more than 60 years old), type of index thrombosis (arterial or venous), and type of treatment after thrombosis: antithrombotic prophylaxis with antiplatelet agents or long-term oral anticoagulation, cytoreduction by HU, or any type of other pharmacological cytoreductive treatment. Statistical analyses were performed using MedCalc Statistical Software version 17.2 (MedCalc Software bvba, Ostend, Belgium; http://www.medcalc.org; 2017).

## Results

### Patient characteristics and rate of recurrent thrombosis

The clinical and laboratory features of the pooled cohort are reported in Table 1. Most patients were affected by PV or ET. About two-thirds of the cohort consisted of patients with arterial thrombosis; the clinical manifestations more represented were cerebrovascular diseases, deep vein thrombosis of the legs with or without pulmonary embolism, and splanchnic vein thrombosis, mirroring the patho-epidemiology of MPN^1,2^. Cytoreduction (mostly with HU) and/or antithrombotic treatment was given to 97.4% of the patients. The dose of HU was given according to the current medical practice, i.e., aimed to control hypercythemia, reducing the cell count within the normal range and/or to recommended values (e.g., Hct ≤ 0.45 in PV patients). The incidence rate of recurrent thrombosis was 5.7% patient-years, as expected from previous estimates^3–7^, with no significant difference between the rate of recurrent thrombosis after a first arterial event or a first venous event (*p* = 0.67) (Table 2). However, the site of the first thrombosis was a strong predictor of the site of recurrence, confirming previous findings^4,5^. Among the patients who had a novel thrombosis after the index event, the coincidental involvement of the same arterial or venous district of the first thrombosis occurred in 77.2% of those with an initial arterial thrombosis (*p* < 0.0001 vs. the rate of novel venous thrombosis) and in 71.8% of those with an initial venous thrombosis (*p* < 0.0001 vs. the rate of novel arterial thrombosis). After adjustment for sex, age, and antithrombotic or cytoreductive treatment, the risk of arterial or venous recurrence was significantly higher in patients having had a first arterial thrombosis (HR 2.94, 95% CI 1.92–4.51, *p* < 0.0001) or a first venous thrombosis (HR 3.30; 95% CI 2.18–5.01, *p* < 0.0001), respectively.

**Table 1:** Clinical features of the cohort at the index thrombosis (*N* = 1500)

Diagnosis	Total – *N* (%)
PV	590 (39.3%)
ET	761 (50.8%)
PMF	149 (9.9%)
Total	1500 (100%)
Male/female	652/848 (43.4%)
Age at thrombosis—median (range)	65 (19–90)
> = 60 years	848 (56.5%)
Type of index thrombosis	
* Arterial thrombosis (N* *=* *935)*	
Acute coronary syndrome	107 (7.1%)
TIA	302 (20.1%)
Ischemic stroke	486 (32.5%)
Other arterial thromboses	40 (2.7%)
* Venous thrombosis (N* *=* *565)*	
DVT of the legs and/or pulmonary embolism	293 (19.5%)
Budd–Chiari syndrome	38 (2.5%)
Portal–mesenteric venous thrombosis	180 (12.0%)
Cerebral vein thrombosis	40 (2.7%)
Other venous thromboses	14 (0.9%)
Aspirin or other antiplatelet agents	892 (59.4%)
Oral anticoagulation (VKA, DOACs) +/− ASA	499 (33.2%)
Hydroxyurea	975 (65.0%)
Hydroxyurea alone	55 (3.7%)
Hydroxyurea + antiplatelet agents	589 (39.3%)
Hydroxyurea + oral anticoagulation	290 (19.3%)
Hydroxyurea + other regimens*	41 (2.7%)
Other cytoreductive drugs	257 (17.1%)

*s.c. heparin, VKA/DOACs + antiplatelet agents, and dual antiplatelet treatment

**Table 2:** Incidence of thrombosis and bleeding after the index event

	Events, *n* (%)	Incidence rate % pt-years (95% CI)
*After a first thrombosis* *(N* *=* *1500, total patient-years* *=* *6075)*		
Thrombotic events	348 (23.2%)	5.7 (5.1–6.4)
Major bleeding	77 (5.1%)	1.3 (1.0–1.6)
*After first arterial thrombosis* *(N* *=* *935, total patient-years* *=* *3907)*		
Thrombotic events	220 (23.5%)	5.6 (4.9–6.4)
Arterial thrombosis	170 (18.2%)	4.3 (3.7–5.0)
Venous thrombosis	50 (5.3%)	1.3 (0.9–1.7)
Major bleeding	44 (4.7%)	1.1 (0.8–1.5)
*After a first venous thrombosis* *(N* *=* *565, total patient-years* *=* *2168)*		
Thrombotic events	128 (22.6%)	5.9 (4.9–7.0)
Arterial thrombosis	36 (6.3%)	1.7 (1.2–2.3)
Venous thrombosis	92 (16.3%)	4.2 (3.4–5.2)
Major bleeding	33 (5.8%)	1.5 (1.0–2.1)

### Overall efficacy of treatments in preventing recurrent thrombosis

A multivariable analysis of the effect of treatments in the overall cohort showed a significantly protective effect either of antiplatelet agents or VKA treatment, with a decreased rate of re-thrombosis by 42%. On the other hand, HU was associated with a reduced risk of only borderline statistical significance (Table 3). However, analyzing the arterial and venous thrombotic outcomes separately, after adjustment for the site of the first thrombosis (arterial vs. venous), antiplatelet and VKA treatment retained their statistical significance in preventing arterial recurrences but also HU resulted in an independent protective factor. On the contrary, antiplatelet agents and HU were less effective in preventing novel venous thromboses (Table 3). Finally, cytoreductive drugs other than HU (i.e., anagrelide, interferon, pipobroman, busulfan, and ruxolitinib) did not affect the rate of recurrent thrombotic events.

**Table 3:** Effect of long-term treatments on the risk of recurrences after the index thrombosis in the entire patient cohort (multivariable analysis).

	Overall recurrent thromboses (HR, 95% CI)		Arterial recurrent thrombosis (HR, 95% CI)*	*p*	Venous recurrent thrombosis (HR, 95% CI)*	*p*
Age > 60 years	1.23 (0.99–1.52)	0.06	1.18 (0.89–1.57)	0.23	1.28 (0.91–1.79)	0.15
Male sex	0.94 (0.76–1.17)	0.60	0.97 (0.73–1.28)	0.99	0.91 (0.65–1.28)	0.61
Antiplatelet treatment	**0.58 (0.43–0.79)**	**0.0005**	**0.54 (0.35–0.82)**	**0.003**	0.64 (0.40–1.03)	0.07
Oral anticoagulation (VKA or DOACs)	**0.58 (0.41–0.81)**	**0.001**	**0.58 (0.35–0.96)**	**0.03**	**0.60 (0.37–0.95)**	**0.03**
Hydroxyurea	**0.75 (0.57–1.00)**	**0.05**	**0.67 (0.46–0.98)**	**0.04**	0.87 (0.56–1.33)	0.52
Cytoreduction with agents other than hydroxyurea^#^	1.04 (0.74–1.45)	0.80	0.94 (0.61–1.46)	0.80	1.22 (0.72–2.04)	0.44

*HR* hazard ratio

*Multivariable analysis adjusted for the arterial or venous site of the first thrombosis

^#^Anagrelide, interferon, pipobroman, busulfan, and ruxolitinib

Bold values are those with statistical significance

### Vessel site-related efficacy of treatments in preventing recurrent thrombosis

The multivariable analysis limited to the patients with first arterial thrombosis confirmed that recurrent arterial thrombosis was prevented by antiplatelet agents (HR 0.49, 95% CI 0.31–0.78, *p* = 0.003) and by HU (HR 0.64, 95% CI 0.42–0.98, *p* = 0.04) and only partially by VKA (HR 0.53, 95% CI 0.27–1.04, *p* = 0.06); on the contrary, in patients with the first venous thrombosis, the venous recurrences were more prevented by VKA (HR 0.57, 95% CI 0.35–0.94) than by antiplatelet agents (0.71, 95% CI 0.41–1.24, *p* = 0.24) or HU (HR 0.75, 95% CI 0.46–1.23, *p* = 0.26).

Notably, analyzing patients with VTE according to the site of thrombosis, HU was confirmed to be without a significant effect on the rate of either recurrent thrombosis or recurrent VTE in 218 patients with splanchnic vein thrombosis (HR 0.81, 95% CI 0.39–1.65, *p* = 0.56, and HR 0.92, 95% CI 0.40–2.13, *p* = 0.85, respectively), after adjustment for age, sex, antiplatelet treatment, VKA treatment, and cytoreductive agents other than HU. On the opposite, when analyzing the 293 patients with VTE at common sites (i.e., deep vein thrombosis of the legs and/or pulmonary embolism), HU was found significantly effective in reducing the rate of either recurrent thrombosis or recurrent VTE (HR 0.56, 95% CI 0.32–0.99, *p* = 0.04, and HR 0.50, 95% CI 0.26–0.95, *p* = 0.03, respectively). Such findings were not substantially modified after adjustment of the model for the white blood cell count at diagnosis (data not shown).

**Fig. 1:**
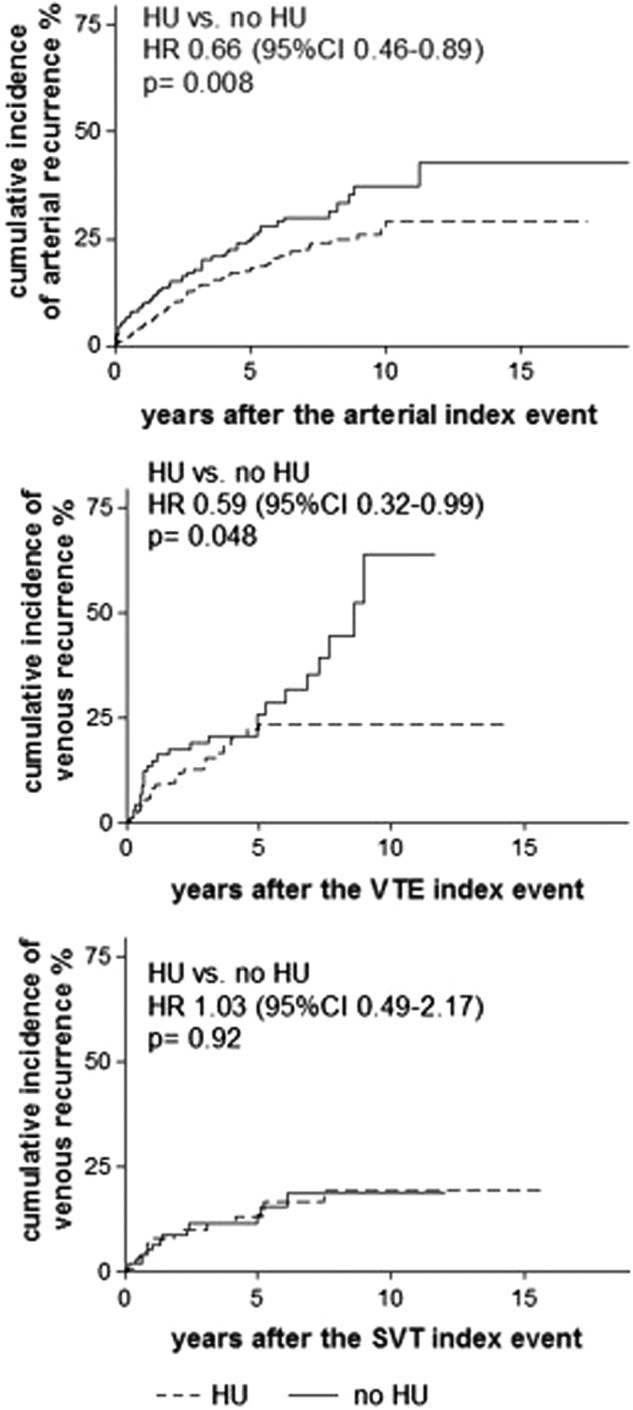
Effect of hydroxyurea (HU) on the cumulative incidence of recurrent arterial thrombosis after an arterial event (top panel) and of recurrent venous thrombosis after a venous thromboembolism (VTE) at common sites (i.e., legs and pulmonary vessels) (middle panel) or after a splanchnic vein thrombosis (SVT) (bottom panel)

Figure 1 shows the favorable cumulative incidence of recurrent thrombosis after the first arterial event in patients receiving HU and no effect of this drug after the first splanchnic venous thrombosis. No significant advantage was observed in prevention of arterial recurrences in the patients with the first arterial thrombosis, taking both HU and antiplatelet treatment over those taking only HU (HR 0.57, 95% CI 0.17–1.29, *p* = 0.14) or only antiplatelet treatment (HR 0.82, 95% CI 0.52–1.17, *p* = 0.26); on the other hand, no significant advantage was observed in prevention of venous recurrences in the patients with the first venous thrombosis taking both HU and VKA over those taking only HU (HR 0.76, 95% CI 0.30–1.82, p = 0.51) or only VKA (HR 0.92, 95% CI 0.55–1.57, *p* = 0.79). The probability of recurrence-free survival after venous VTE at common sites was not seen in the first 5 years of HU treatment (HR at 5 years 0.71, 95% CI 0.36–1.27, for HU vs. no HU, *p* = 0.23), while a positive effect was documented in the 76 patients who were exposed to this drug for more than 5 years. In these cases, recurrent VTE occurred in 1/48 patients on HU and in 7/28 patients not receiving this drug (HR 0.08, 95% CI 0.02–0.46, *p* = 0.0028). The patients with a follow-up shorter or longer than 5 years did not differ as regards the rate of treatment with HU only (*p* = 0.86), HU and VKA (*p* = 0.35), and VKA only (*p* = 0.52).

## Discussion

We confirmed that in MPN patients, the rate of recurrent thrombosis is high and occurs in 5.7% patient-years in spite of secondary prophylaxis administered by specialized centers^4–8^. Cytoreductive treatment with HU is strongly recommended as first-line therapy in high-risk patients, particularly in those with a history of thrombosis^14^. However, whether HU has a different protective role on the prevention of arterial or venous thrombosis is uncertain and was addressed in a limited number of studies^5,10,13^.

In the present study, involving a huge number of 1500 patients with arterial or venous events, multivariable analysis demonstrated a different protective action of HU on recurrences according to the district in which the first episode of thrombosis occurred. We confirmed here that HU in association with both antiplatelet and VKA treatment, was able to reduce the rate of recurrent arterial thrombosis, but we did not show a significant action of the drug in the prevention of total recurrent venous thrombosis after thrombosis at common sites and splanchnic vein thrombosis.

Although venous and arterial thrombotic disease have been historically regarded as distinct diseases with differing etiologies, these two classes of thrombotic events share common characteristics. Both hypercoagulability and inflammation contribute to the development of arterial and venous thrombi, and risk factors for the two diseases are not altogether dissimilar^15^. However, the magnitude of arterial risk associated with a previous arterial thrombosis is not as high as that associated with a previous venous thrombosis and vice versa, so that the etiology of these two thrombotic diseases may be distinct.

Arterial and venous thrombosis can develop through different cellular and plasma pathways^16^, so that it is not surprising that drugs with different targets can achieve different results in preventing thrombosis in different vessel districts. The prevention of arterial recurrences mediated by HU may be due to myelosuppressive activity but also to the direct antithrombotic action on polymorphonuclear leukocytes function and their interaction with platelets^17^. The importance of leukocytes in the pathophysiology of arterial thrombosis is well established in non-MPN individuals^18,19^. Accordingly, leukocytosis has been reported as a potential determinant for myocardial infarction both in PV^20^ and in ET^21,22^ but not for VTE^20–22^.

On the other hand, activation of the coagulation system is the primary cause of venous thrombosis, and precedes platelet activation and aggregation. This may explain why HU had a limited activity in preventing recurrences of common venous thromboses in MPN patients, while anticoagulation therapy independently resulted as a protective drug reducing the rate of recurrences, as in the non-MPN population. In the latter non-MPN subjects, the risk of recurrence after unprovoked VTE is the highest during the first 5 years after discontinuation of anticoagulation and then declined, but never completely disappeared^23^. In our study, the efficacy of HU in patients with VTE at common sites was not demonstrated in the first 5 years after the incident event, suggesting that in this period, the cellular component of venous thrombogenesis is not as important as plasma hypercoagulability, which is well controlled by oral anticoagulants. Subsequently, HU showed a strong positive effect, indicating an advantage in the control of myeloproliferation.

Of note, we failed to show a positive action of HU in preventing recurrences after the first incident episode of splanchnic vein thrombosis. The reason of this finding is difficult to explain; it could be speculated that in patients with splanchnic vein thrombosis, hypercythemia is less frequent^24^, so that cytoreduction in this setting could be less crucial than otherwise. Moreover, given the unique environment of the splanchnic venous system, it is likely that the mechanisms of SVT formation differ from the mechanisms of arterial and VTE at common sites. Liver endothelial cells isolated from two patients with Budd–Chiari syndrome^25^ and spleen endothelial cells^26^ have been found to carry the JAK2 V617 mutation, so that aberrant endothelial cells may eventually contribute to SVT pathogenesis. Therefore, the occurrence of SVT, occurring either as the heralding presentation of MPN or as a complication during the course of the disease, poses an important clinical decision on the use of cytoreduction. We suggest to prescribe HU only in the presence of hypercythemia or in the case of progressive disease.

In our analysis, VKA were significantly effective in reducing the rate of venous thrombotic recurrences by 40%. This reduction in risk is comparable with the results obtained in the prevention of recurrent VTE in the non-MPN population^27^. However, given the different risk baselines in the MPN and non-MPN patients, the absolute incidence rate of recurrent venous thrombosis in MPN patients remains higher than that observed in non-MPN patients treated with VKA, as previously reported^7^.

Finally, in the patients treated with cytoreductive drugs other than HU (i.e., anagrelide, busulfan, pipobroman, interferon, and ruxolitinib), we did not show any antithrombotic efficacy in terms of reduction of recurrences. However, this finding should be taken with caution, because the number of treated patients for each drug is likely not enough powered.

In conclusion, although our study has limitations due to the retrospective nature, this series of 1500 MPN patients with incident index thrombosis is the largest ever reported, so that statistical power is adequate. The different effects of HU on the protection of arterial or venous vessels likely mirror the different pathogenetic mechanisms involved in thrombosis of different districts. The results obtained by VKA for secondary prophylaxis of recurrent VTE although significant are not entirely satisfactory. Overall, we conclude that our therapy armamentarium to prevent arterial and particularly venous recurrences in MPN is suboptimal, and future studies to optimize and personalize the secondary pharmacological prophylaxis are urgently warranted.
